# In situ lymphoma imaging in a spontaneous mouse model using the Cerenkov Luminescence of F-18 and Ga-67 isotopes

**DOI:** 10.1038/s41598-021-03505-3

**Published:** 2021-12-14

**Authors:** Zsombor Ritter, Katalin Zámbó, Péter Balogh, Dávid Szöllősi, Xinkai Jia, Ákos Balázs, Gabriella Taba, Dániel Dezső, Ildikó Horváth, Hussain Alizadeh, David Tuch, Kunal Vyas, Nikolett Hegedűs, Tibor Kovács, Krisztián Szigeti, Domokos Máthé, Erzsébet Schmidt

**Affiliations:** 1 Department of Nuclear Medicine, Medical School, University of Pécs, Pécs, Hungary; 2 Department of Immunology and Biotechnology, University of Pécs, Pécs, Hungary; 3 Department of Biophysics and Radiation Biology, Semmelweis University Faculty of Medicine, Budapest, Hungary; 4 Semmelweis University Faculty of Medicine 1st Department of Surgery, Budapest, Hungary; 5 Semmelweis University Dosimetry and Medical Physics Service, Budapest, Hungary; 6 1St Department of Internal Medicine, University of Pécs Medical School, Pécs, Hungary; 7 Lightpoint Medical Ltd., Rickmansworth, UK; 8 CROmed Ltd., Budapest, Hungary; 9 Institute of Radiochemistry and Radioecology, University of Pannonia, Veszprém, Hungary; 10 In vivo Imaging Advanced Core Facility, Hungarian Center of Excellence for Molecular Medicine (HCEMM), Budapest, Hungary

**Keywords:** Cancer imaging, Cancer metabolism, Cancer microenvironment, Cancer models, Haematological cancer, Tumour heterogeneity, Molecular medicine

## Abstract

Cerenkov luminescence imaging (CLI) is a promising approach to image-guided surgery and pathological sampling. It could offer additional advantages when combined to whole-body isotope tomographies. We aimed to obtain evidence of its applicability in lymphoma patho-diagnostics, thus we decided to investigate the radiodiagnostic potential of *combined* PET or SPECT/CLI in an experimental, novel spontaneous high-grade B-cell lymphoma mouse model (Bc.DLFL1). We monitored the lymphoma dissemination at early stage, and at clinically relevant stages such as advanced stage and terminal stage with in vivo 2-deoxy-2-[^18^F]fluoro-d-glucose (FDG) positron emission tomography (PET)/magnetic resonance imaging (MRI) and ^67^Ga-citrate single photon emission computed tomography (SPECT)/MRI. In vivo imaging was combined with ex vivo high resolution CLI. The use of CLI with ^18^F-Fluorine (F-18) and ^67^Ga-Gallium isotopes in the selection of infiltrated lymph nodes for tumor staging and pathology was thus tested. At advanced stage, FDG PET/MRI plus ex vivo CLI allowed accurate detection of FDG accumulation in lymphoma-infiltrated tissues. At terminal stage we detected tumorous lymph nodes with SPECT/MRI and we could report in vivo detection of the Cerenkov light emission of ^67^Ga. CLI with ^67^Ga-citrate revealed lymphoma accumulation in distant lymph node locations, unnoticeable with only MRI. Flow cytometry and immunohistochemistry confirmed these imaging results. Our study promotes the combined use of PET and CLI in preclinical studies and clinical practice. Heterogeneous FDG distribution in lymph nodes, detected at sampling surgery, has implications for tissue pathology processing and it could direct therapy. The results with ^67^Ga also point to the opportunities to further apply suitable SPECT radiopharmaceuticals for CLI.

## Introduction

Haematological malignancies can occur at various life stages throughout age groups. The most frequent lymphoid malignancy in adulthood is diffuse large B-cell lymphoma (DLBCL). Adult DLBCL occurrences make up approximately 30–40% of all lymphoid malignancies. Around half of the morbidities occur in the age group of patients older than 60 years^1–3^. Despite well-established therapy protocols, 30% of patients are resistant to standard chemo-immunotherapy mostly due to heterogeneous origins of the disease.

Diffuse large B-cell lymphomas are a particularly heterogeneous group of malignant lymphoproliferative neoplasms^2,3^ and for an effective therapeutic strategy it is important to understand their heterogeneous phenotypes. When only histopathological markers are used, germinal centre (GC) or non-GC phenotypes are distinguished. By genetic marker analysis of the cell of origin (COO), the other main DLBCL group besides GC is the activated B-cell (ABC) subtype. Patients within the ABC-COO group of DLBCL usually have lower clinical response rate and worse prognosis^4–6^. In recent years, new DLBCL subgroups have been created to provide more accurate classification and more accurate prognosis prediction with regards to response to therapy, based on special molecular genetic differences^5,6^ exploiting the possibility of detailed genomic characterization with next generation sequencing^7,8^.

In connection with these recent advances in lymphoma therapy, imaging-based diagnostic tools have also evolved. The application of new in situ in vitro imaging holds the promise of timely access to advanced differentiation of DLBCL patient clusters. The so identified subgroups of patients would then benefit from quick and tailored immune- or intensive chemotherapy.

Clinical management of the disease involves the evaluation of enlarged lymph nodes, starting with cytologic aspiration sampling. However, aspiration samples are usually not definitive in diagnostics, much less for appropriate selection for any subgroup-specific therapeutic protocol. Before starting definitive therapies, further excisional biopsy and detailed histological analysis of at least one accessible, tumorous part of a suspect lymph node is a mandatory step in the course of diagnostics^9,10^.

At present 2-deoxy-2-[18F]fluoro-d-glucose (FDG) positron emission tomography/X-ray computed tomography (PET/CT) (hereinafter referred to as FDG PET/CT) is the most widespread diagnostic modality for patient staging and monitoring therapy^9–14^ in high-and middle-high income countries.

In routine diagnostics, the Deauville score is used to evaluate interim and restaging patient FDG PET/CT results^11–13^. In addition to the Deauville score, heterogeneity and a number of other semi-quantitative parameters (including maximal standardized uptake value (SUV MAX), total lesion glycolysis (TLG), and metabolic tumor volume (MTV) can also be used. Their prognostic significance has been intensively studied^14–18^ but no definitive clinical metric proposal has been formed yet.

On the other hand -before the widespread availability of FDG- earlier clinical practice in diagnosis and therapeutic management of lymphomae extensively relied on gallium [^67^Ga]; 2-hydroxypropane-1,2,3-tricarboxylate (^67^Ga-citrate) single photon emission computed tomography (SPECT) and two-dimensional gamma scintigraphy with the same isotope. The diagnostic performance of FDG is superior than that of ^67^Ga-citrate, however the relevance of ^67^Ga imaging is somewhat still present in lymphoma. In addition to lymphoma diagnostics, ^67^Ga is also increasingly viewed as a therapeutic isotope in oncology by virtue of its Auger-electron emission and specific lymphoma uptake.

By localising involved lymph nodes, subsequent sampling could benefit from the use of the currently obligatory pre-therapy FDG PET/CT or ^67^Ga-citrate scan images to establish therapeutic directions. As an example, identifying, removing and analysing lymph nodes and inhomogeneous tumor masses using the available Cerenkov light emission of Fluorine-18 isotope after the PET/CT exam would lead to more precise molecular biology evaluation, hence leading to choosing a better therapeutic protocol^17–19^.

In this direction, our group has previously described the Bc.DLFL1 tumor model as a spontaneous high-grade lymphoma isolated from BALB/c mice. This model shows preferential tissue distribution to mesenteric lymph nodes and spleen during peritoneal spreading, and propagates via the lymphatic vessels^20^. This well-characterized lymphoma is a suitable model for preclinical investigations, including assessment of novel imaging techniques^21^. The model invariably leads to death 21 days after the animals are inoculated, with 90% of animal deaths occurring between 11 and 14 days. Thus, animals are considered to be in the end-stage from day 11 post intraperitoneal inoculation.

The basic aim of the present study was to assess staging possibilities by imaging and monitoring dissemination of Bc.DLFL1 lymphoma in vivo with PET and single photon emission computed tomography (SPECT) and ex vivo with high resolution Cerenkov luminescence imaging (CLI). CLI as an advanced imaging technique^22,23^ has a considerable potential for clinical translation. Every new CLI application is of high medical interest, especially those using clinically authorised and marketed radiopharmaceuticals or isotopes. One such yet not reported isotope in practical CLI is ^67^Ga. However underreported, theoretical calculations show that the high energy gamma photons emitted during the decay of this isotope could lead to secondary electrons resulting in Cerenkov luminescence in water or in water-containing tissues^24–26^. Therefore, we assessed CLI combined with the usual FDG positron emission tomography/magnetic resonance imaging (PET/MRI) and gallium [^67^Ga]; 2-hydroxypropane-1,2,3-tricarboxylate (^67^Ga-citrate) single photon emission computed tomography combined with magnetic resonance imaging (SPECT/MRI) imaging, too. We also investigated whether the use of a CLI device could help to monitor the spreading of the DLBCL model ex vivo and if this CLI device could detect details of metastatic foci. Animals were imaged in three tumor stages for both radiopharmaceuticals: at an early stage of 4 days post inoculation, in the advanced stage at 8 days and in terminal stage. CLI was aimed to direct the search for small or heterogeneous tumorous foci. Using this imaging sequence, the presented methodology of tailored ex vivo selection and histopathologic processing of tumor volumes can also progress towards clinical translation.

## Materials and methods

### Experimental animal model of Bc.DLFL1 lymphoma propagation

All studies reported here have been carried out in accordance with the rules on animal welfare and regulations on other respective subjects in vigour in Hungary. The study had been designed and performed in compliance with the ARRIVE guidelines. The study was approved by the Ethics committee of the University of Pécs. Furthermore, all procedures involving live animals were carried under permission of the National Food Chain Safety Office of Hungary, Department of Animal Health, under license number BA02/2000-16/2015. BALB/c mice bred at the SPF Animal Breeding Unit of the University of Pécs, Department of Immunology and Biotechnology, aged between 8 and 12 weeks, were used as lymphoma recipients. After retrieval, the mice were adapted to conventional animal facility of the Department of Immunology and Biotechnology. The Bc.DLFL1 lymphoma cells were maintained as serial intraperitoneal passage.

### Grouping and number of animals by tumor stage

In both the FDG PET and the ^67^Ga-citrate SPECT/MRI experiments animals were grouped into early stage (imaging 4 days post inoculation of lymphoma cells), advanced stage (imaging 8 and 9 days post inoculation) and terminal stage (imaging 11 and 12 days post inoculation). (Altogether 36 mice at inoculation were grouped into six groups with six mice per group.) Each stage groups contained n = 6 animals per radiopharmaceutical. However, in the end-stage DLBCL group of animals at 11 days post inoculation, deaths occurred thus five animals were amenable to the combined in vivo tomographic—ex vivo CLI imaging from the FDG PET group, and one animal was usable for the ^67^Ga-citrate SPECT/MRI imaging from its group of originally six animals.

### In vivo imaging using [18F]FDG PET/MRI

Each animal received between 10 and 15 MBq of FDG (Pozitron-Scan, Pozitron, Hungary) i.v. in a 0.1 mL lateral tail vein injection 1 h before the PET and 1.5 h before the subsequent CLI imaging. Animals were fasted for 8 h before scans. PET and MRI images were acquired in a nanoScan 1 Tesla PET/MRI system (Mediso Ltd, Hungary) and in a Micropet P4 (Concorde Microsystems, US) small animal PET system. Static scans of 15-min duration were collected 60 min post injection in an energy window of 350–750 keV. All PET images were reconstructed using the respective device’s three-dimensional Maximum A Posteriori (MAP, P4 system) or three-dimensional Monte-Carlo modeled Ordered Subsets Expectation Maximisation (Tera-Tomo 3D-OSEM, Mediso system) algorithm, with corrections for dead time, scatter, and attenuation where available. Reconstructed images of 1 mm voxel size were then visualised using the VivoQuant (inviCRO, USA) and Fusion (Mediso, Hungary) softwares.

All MR images were acquired in a Mediso nanoScan 1T MRI with a three-dimensional T1-weighted gradient echo sequence, with 300 micron voxel size, 6 excitations, 12.1 ms/2.9 ms Transmit/Receive times, and 15 degrees of flip angle.

### In vivo imaging using Ga-67 citrate SPECT/MRI

For SPECT, 4.1–5.4 MBq of ^67^Ga-citrate (CIS Bio, France) was injected iv. in 0.2 mL 24 h before imaging A 45-min SPECT scan was then performed using a NanoSPECT/CT Silver Upgrade small animal SPECT system (Mediso Ltd, Hungary) using both the 93 keV and 184 keV photopeaks in a 20–20% energy window, with 60 acquisition views and a Monte–Carlo model based three-dimensional SPECT reconstruction (Tera-Tomo, Mediso Ltd, Hungary) resulting in an image volume of 0.6 mm voxels. The same animal in the same animal bed allowing constant position was then immediately subjected to a whole-body three-dimensional MR imaging sequence in the nanoScan 1T MRI subcomponent. After these acquisitions, tissues were examined with ex vivo CLI, histology and immunohistochemistry following the in vivo imaging. All live animal image acquisitions were performed under 2% isoflurane anesthesia in medical oxygen in a special cross-compatible animal holder bed (MultiCell, Mediso, Hungary) which kept the animals immobilised during cross-modality and cross-device scans.

### Processing of ex vivo tissues for CLI and subsequent histology in situ

The entire gastrointestinal tract (between the subphrenic segment of oesophagus and the upper third of rectum together with the adjoining mesentery) was removed and placed in a 10 cm Petri dish, with the intestinal folds flattened. Under gentle pressure the dissected gut-mesentery complex was fixed in cold 4% buffered paraformaldehyde for 15 min, followed by repeated rinsing with PBS. The entire complex was stained with Mayer’s hematoxylin solution for 10 min, followed by blue stain intensification in tap water for 5 min.

### CLI imaging

Following the end of PET acquisitions, Cerenkov light imaging of the Cerenkov luminescence produced in the tissues containing high concentrations of the injected radionuclidic tracer, was performed using a prototype high resolution Cerenkov imaging system with an exceptionally sensitive imaging chain. (LighPath, Lightpoint Medical, UK) Tissue specimens were placed in the sample holder of the instrument and image acquisition was performed over them for 10 min of signal integration, with 4 × 4 pixel binning in 512 × 512 pixels, with a 0.15 mm pixel size of the imaging chain in a field of view of 8 × 8 cm. White light photographic background was also collected by the instrument for anatomical reference. Images of Cerenkov Luminescence and the background white-light images were exported to DICOM format by the instrument’s dedicated software. Further two-dimensional rigid co-registration, post-processing with a median filter of 3 pixels kernel, rotation and color look-up table changes were done using Fusion (Mediso, Hungary) and VivoQuant (inviCRO, USA). Images were then visualized with a unified common colour Look-Up Table (LUT) across animals, with arbitrary units of light intensity based on the emCCD detector pixel counts overlaid on the white-light photographs.

The clinical prototype LightPath system used in the study had, under the same detector pixel binning condintions as mentioned above, a minimum detectable activity of 20 kBq in a scan time of 100 s and a volume of 0.02 mL for ^18^F in a 0.315 mm radius microwell. During the evaluation of system performance for setting up the ^67^Ga study, we measured a minimum detectable activity of 300 kBq of ^67^Ga isotope, in an equivalent scan time for a similar well, volume of 0.02 mL with the same pixel binning settings. The performance test results obtained from the manufacturer of the prototype system indicated an intrinsic resolution of the imaging chain of 0.93 ± 0.06 mm by a proprietary analysis of the line spread function at full width half maximum.

### Histology and immunofluorescence

Those lymph nodes that have been identified as tumorous, and all spleens, were processed for haematoxylin–eosin (H&E) histology. They were fixed in 10% buffered formaldehyde using standard histological protocols. Immunofluorescence for lymphoma identification was performed with cryostat sections from tissue blocks embedded in OCT medium (BioOptica, Italy) using FITC-conjugated rat anti-mouse IgM (clone B7.6) and Alexa Fluor 647-conjugated rat anti-mouse B220 (clone RA3-6B2) monoclonal antibodies produced at the Department of Immunology and Biotechnology, University of Pécs. The sections were and analysed using BX61 Olympus microscope using the AnaliSys software.

### Flow cytometry

Mesenteric lymph nodes of lymphoma-bearing mice were collected and crushed between two frosted ends of histological glass slides. The cells were filtered through 70 µm cell strainers and centrifuged. After resuspension in PBS-0.1% BSA-0.1% Na-azide buffer, the cells were incubated with FITC-conjugated anti-mouse IgM and Alexa Fluor 647-conjugated anti-B220 mAbs. Lymphoma cells and residual lymphocytes were distinguished by gating on forward and side scatter (FSC/SSC) parameters related to size and granularity, respectively, using a BD FACSCalibur flow cytometer and CellQuest Pro software (both BD Biosciences, UK).

## Results

### Early stage DLBCL escapes radioisotopic whole-body and tissue-level detection with PET, SPECT or CLI

Every animal in each group of the early stage FDG group and also in the early and advanced stage ^67^Ga-citrate group could be tested for signal capture and levels in tomographic in vivo and Cerenkov Light imaging. No circumscribed tumor uptake was seen over the background with either FDG or ^67^Ga-citrate in early stage animals (n = 3 for FDG, n = 3 for ^67^Ga-citrate). Using ^67^Ga-citrate, no clearly distinguishable SPECT foci nor visibly tumor-associated Cerenkov signal was seen in advanced-stage animals either.

^67^Ga-citrate SPECT and subsequently ^67^Ga-citrate CLI detected lymphoma only at its terminal, very advanced stage. However, a well identifiable ^67^Ga-Cerenkov light signal was captured in the physiologically Gallium-avid regions of the mouse intestines in all imaged animals regardless of tumor stages. In the case of the advanced (8 days post inoculation) stage lymphoma SPECT and CLI images with ^67^Ga-citrate, high background bowel activity and consequent CLI signal hampered the distinguishing of tumor signal from background CLI luminescence of ^67^Ga in mesenteric and abdominal scans, thus interpretation of images to tumorous or non-tumorous uptake was not possible (data not shown).

### Characterization of [18F]FDG uptake is feasbile both in vivo with PET/MRI and ex vivo with CLI in advanced stage lymphoma

**Figure 1:**
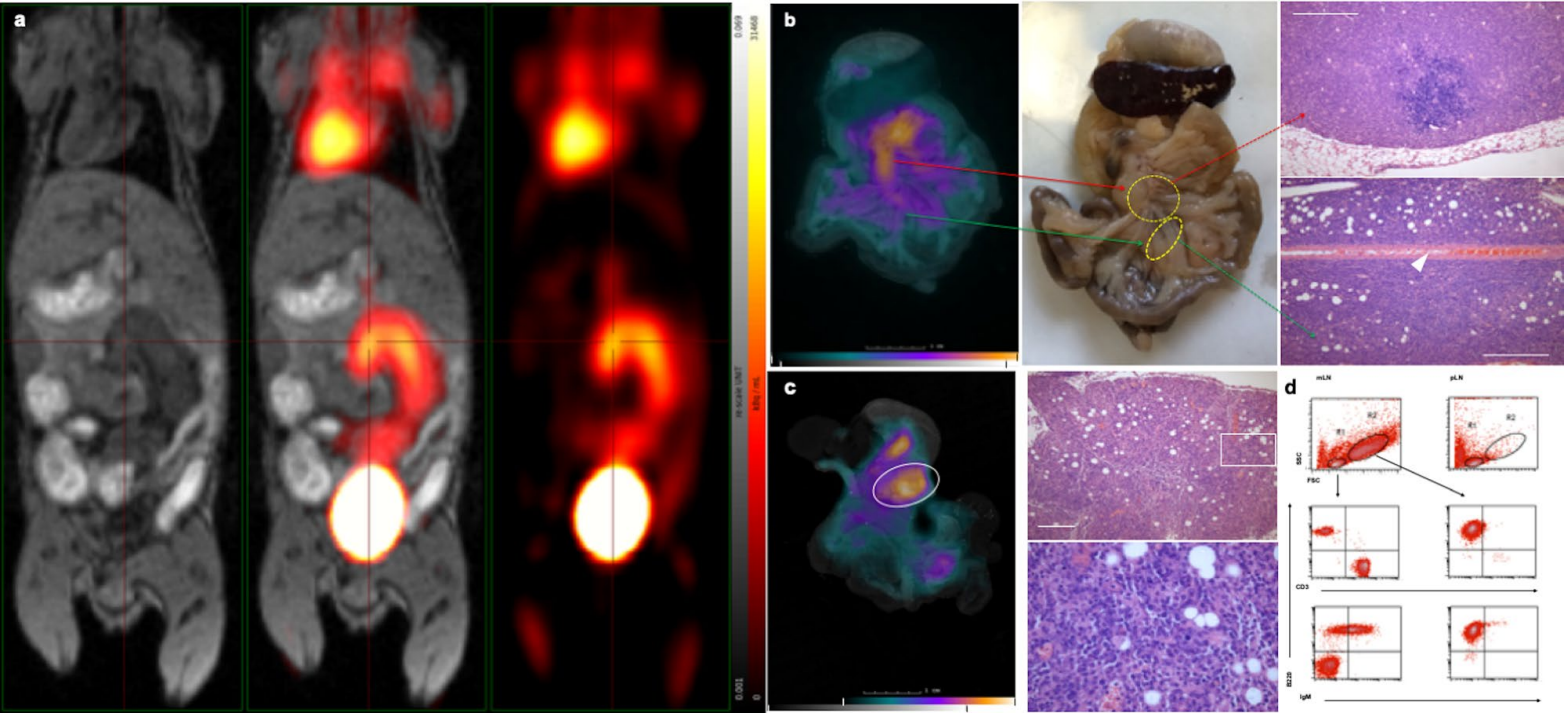
Molecular imaging results in advanced stage lymphoma-bearing mice. (**a**) PET/MR image in advanced stage: FDG-avid enlarged mesenteric lymph nodes are visible (horizontal plane, representative image). (**b**) Colour overlay of Cerenkov light emission (CLI) on the white light photograph of the intestinal preparation, with the spleen at the top of the image. FDG-avid lymphomatous regions and foci in the peritoneum and omentum are coloured orange. The H&E staining (upper right) depicts the diffuse infiltration of the omentum (scale bar = 200 µm), in the area marked by an ellipse on the CLI picture (scale bar = 200 µm). Lower-right image with high-power magnification shows lymphoblastic dominance corresponding to the area marked with rectangle (scale bar = 50 µm). Representative images from a cohort of mice with *n* = 3. (**c**) Ex-vivo CLI in late stage lymphoma: FDG-avid Bc-DLFL.1 lymphoma foci with H&E staining in mesenteric lymph node (top) and mesenterium (bottom) CLI (left) and, Stereomicroscopic picture of the intestinal preparation (middle), Histology (right): the red arrow shows that the upper encircled area in the stereomicroscopic picture emits Cerenkov light and corresponds to the mesenteric lymph node complex, containing a massive lymphoma infiltrate demonstrated on the top right image (connected by dashed red arrow). The bottom green arrow connects one branch of the mesentery with lesser Cerenkov signal intensity (marked with ellipse), with an extensive lymphoma infiltrate surrounding the mesenteric artery (arrowhead). Scale bar = 200 µm. Representative images from a cohort of mice with *n* = 3. (**d**) Flow cytometric analysis of enlarged mesenteric lymph nodes (mLN) reveals residual small-sized (in R1 gate) CD3-positive T cells and B220-positive B cells (left, middle), latter cells mostly co-expressing IgM (left, bottom), and large number of blast-sized cells (in R2 gate) displaying B220 B-cell marker and absence of CD3-positive cells (right, middle), but lacking surface IgM and CD3 (right, bottom). Lymphocytes from peripheral lymph nodes (pLN) do not contain a distinguishable blast population within the R2 gate (right, top). Representative images from a cohort of mice with *n* = 3.

Using in vivo PET/MRI we found that in an advanced lymphoma stage (at or beyond day 8 post-injection) the infiltrated mesenteric lymph nodes clearly showed a significant accumulation of [18F]FDG following intravenous administration (Fig. 1a). With CLI we observed [18F]FDG accumulation in the infiltrated adipose tissue along the mesenteric vessels, within the enlarged mesenteric lymph nodes, in the omentum and also in the splenic hilus. Subsequent histological analysis of the [18F]FDG signal-positive regions confirmed large infiltrates of lymphoma cells at these sites (Fig. 1b,c). For the quantitative assessment of the degree of lymphoma infiltration, flow cytometric analysis was performed. Lymphoma cells could be distinguished using forward and side scatter (FSC/SSC) parameters reflecting size/granularity characteristics, with the lymphoma cells displaying substantially larger size. However, even at the advanced stage of lymphoma, some residual lymphocytes within the affected mesenteric lymph nodes could still be detected, including both CD3-positive T cells and B220-positive normal B cells, latter population also displaying surface IgM. On the other hand, the infiltrating lymphoma cells express only B220, confirming their DLBCL characteristics (Fig. 1d).

### Ex vivo CLI discerns tumourous infiltrate within lymph nodes identified as distant metastases using Ga-67 citrate SPECT/MR

**Figure 2:**
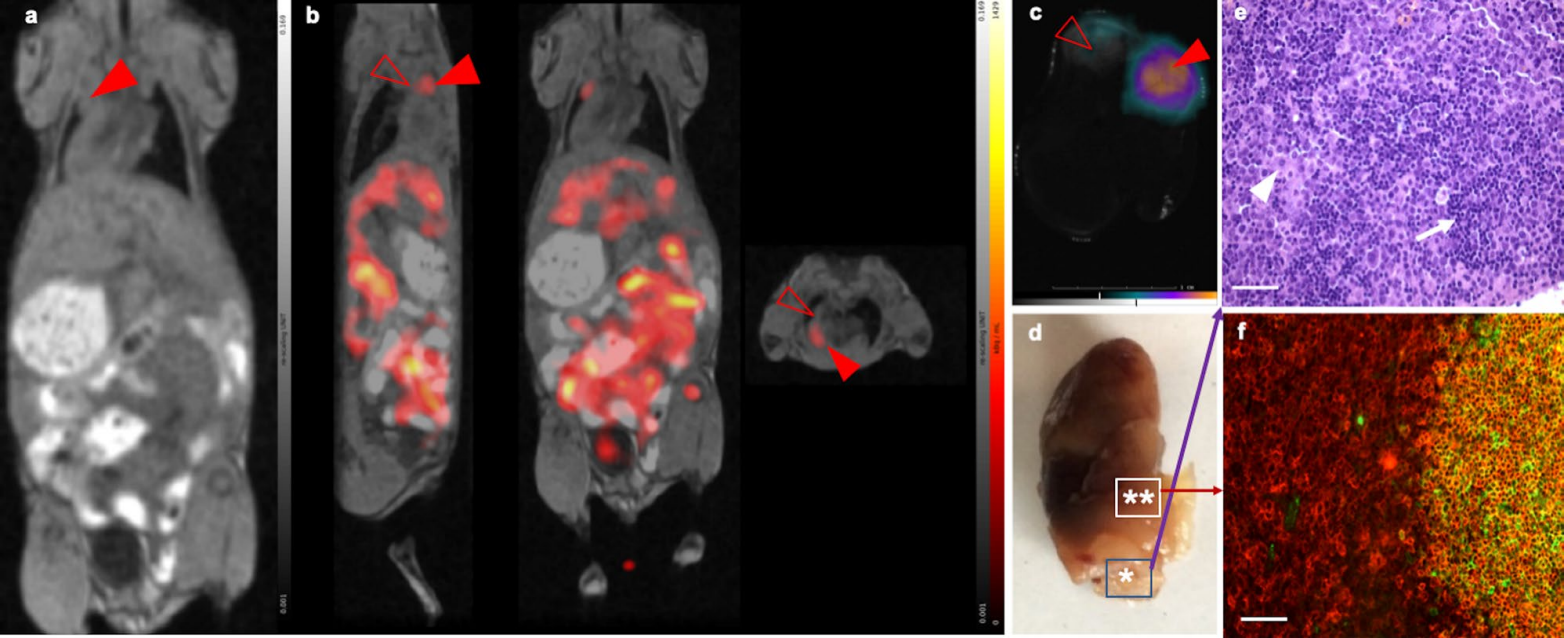
End-stage lymphoma-bearing mouse images show specific accumulation of ^67^Ga-citrate in parathymic lymph nodes. (**a**) MRI: enlarged mediastinal lymph node conglomerate with signs of tumor infiltration (red arrowhead). (**b**) ^67^Ga-citrate SPECT/MRI: SPECT/MRI assessment shows tracer accumulation in a part of the left parathymic lymph node (from left to right in this panel, sagittal, horizontal and axial plane view, focused on the parathymic region). The red full arrowhead shows the tracer accumulating part of the infiltrated lymph node, while the empty arrowhead points to the part of the enlarged lymph node not accumulating ^67^Ga-citrate. (**c**) Ex-vivo Cerenkov image shows ^67^Ga-citrate accumulation in the previously identified part of the parathymic lymph node and low accumulation in the part identified with the empty arrowhead in SPECT/MRI. Scale bar corresponds to 1 cm. (**d**) Stereomicroscopic image of the same parathymic lymph node. Two areas are identified, * in the lower rectangle shows the tumor-infiltrated part, its histology analysis image indicated by a purple arrow, ** in the upper rectangle identifies the tumor infiltration border area in the lymph node. The corresponding immunohistochemistry image in (**f**) is pointed at with a red arrow. (**e**) Haematoxylin–eosin staining microscopy image of a section from the infiltrated part of the parathymic lymph node, identified with (**a**) * in (**d**). The sample is taken from the area in the lower rectangle of the stereomicroscopic image shown with *. Small clusters of residual normal lymphocytes (arrow) are visible, surrounded by large lymphoma infiltrates composed of irregularly shaped lymphoblasts (arrowhead). (**f**) Immunofluorescence histological image from the transitional area of the lymph node labeled **, delineated in the upper rectangle of the stereomicroscopic image in (**e**) demonstrates the remnant of original follicle composed of IgM/B220 double positive B cells adjacent to B220 single positive DLBCL cells (green: IgM, red: B220). Scale bar = 100 µm.

At the advanced stage, the enlarged mesenteric lymph nodes (Fig. 2a) are well detectable using MRI. The distinction of ^67^Ga-citrate accumulation signals in the lymph nodes based on the SPECT alone without MRI would be difficult due to the high background activity caused by the intense non-specific intestinal accumulation of the tracer. On the other hand, the mediastinal region lacks this background. This allows specific detection of distant manifestations with SPECT, even in tissues without obvious MR alterations. Here the SPECT assessment could clearly reveal lymphoma accumulation in the region of parathymic lymph nodes, which were not well noticeable using only MRI analysis in the end-stage lymphoma-bearing mouse (Fig. 2b). The presence of lymphoma infiltrate in parathymic lymph nodes was verified following their removal by using ex vivo CLI (Fig. 2c) and subsequent histological analysis, including dual immunofluorescence labelling for IgM and B220. This approach allows the identification of lymphoma cells by the lack of surface IgM and the preserved expression of B220 marker, thus distinguishing the lymphoma cells from the B220+/IgM+ normal B cells (Fig. 2d–f).

## Discussion

Over the past several decades FDG PET/CT has become the first line tool for the staging and monitoring of therapeutic efficiency in patients with high-grade malignant lymphomas. However no definite diagnosis and well directed therapy can be made in these patients, without a full biopsy of tumorous tissues, as cytology and fine needle aspirations are mostly inconclusive. A medical need for a sampling method also arose to identify a tumorous lymph node in a pre-planned manner with PET/CT images as basis. Tumour-infiltrated lymph node parts should be immediately localised and their targeted biopsy should be performed. In addition, the current sensitivity and resolution achievable by clinical FDG PET are not adequate for monitoring fine details of tracer distribution within foci smaller than approximately 10 mm^27–29^. Other investigative methods such as autoradiography, fluorescence or bioluminescence imaging do not fit to any practical clinical context or they are simply not feasible in humans. Consequently, a sensitive, translatable sampling method applying the gold standard FDG tracer for DLBCL staging and therapeutic feedback remains a clinically important need. This development is effectively assisted by translational pre-clinical models and validation of improved detection methodologies. An ex vivo imaging measurement of metabolic heterogeneity in tumour tissue, in turn, could influence therapy planning, too. In our present work, we provide evidence for the potential radio-diagnostic application of the *combination* of PET imaging with ex vivo Cerenkov luminescence in an experimental high-grade B-cell lymphoma model. This combination provides a more detailed structural assessment of lymph nodes infiltrated by lymphoma cells at a more effective detection sensitivity than any currently available in vivo tool, potentially also offering a much faster evaluation than via traditional processing by tissue histopathology^30,31^.

In addition, this model potentially allows the monitoring the effect of various experimental therapeutic approaches including measurements of metabolic heterogeneity of the tumour cell clusters within lymph nodes quickly after sampling. Combination of PET, SPECT, and CLI allows for this targeted sampling and detailed ex vivo testing. CLI can be used to examine several radioisotopes that are routinely used in the traditional clinical nuclear medicine toolkit^32–34^.

The previously described, well-characterized high-grade mouse B-cell lymphoma model in our work has a restricted, stepwise in vivo propagation from the peritoneal cavity towards the environs of mesenteric lymph vessels into the mesenteric lymph nodes and then the spleen^21^. This allowed us to image different locations of tumor spreading by using our proposed methodology.

We performed a multi-modal multi-tracer whole body in vivo imaging in a clinically relevant advanced stage of the animals with Bc.DLFL1 lymphoma load. This stage of the tumours was then imaged ex vivo using CLI, and the image details were compared to immunohistochemistry and microscopic results in the same animals. We decided to investigate ^67^Ga-citrate, too, as (1) this radiotracer is still the most specific and sensitive means for SPECT imaging of lymphoma^33^, (2) modeling studies have shown an available Cerenkov luminescence from tissue ^67^Ga distribution^24,25,28^ (3) there is a current re-emerging interest in ^67^Ga as an Auger emitter’s therapeutic relevance^35^.

To monitor the in vivo expansion of high-grade lymphoma, we tested whether the in vivo PET/MR and ex vivo CLI modality would allow lymphoma detection. We found that we could detect [18F]FDG accumulation in the organs infiltrated with lymphoma after intravenous administration of [18F]FDG and subsequent in vivo PET/MR and ex vivo CLI. This occurred at and after the advanced stage of lymphoma growth. Our ex vivo results suggest a new clinical application field of [18F]FDG detection using CLI for localisation and observation of the detailed metabolic heterogeneity of the affected lymph nodes^33,36–41^.

The current evaluation of lymphoma expansion and therapy effects in patients relies on the use of Deauville scoring, using hepatic FDG uptake and mediastinal blood pool as reference value^11,13,14^ and several semiquantitative parameters^15–17^. To optimize therapy and plan for clinical monitoring, we propose that—besides Deauville scoring and the currently studied PET derived semiquantative parameters—it would be particularly important to obtain information about the heterogeneity of individual enlarged, tumorous lymph nodes. Measurement of glycolytic heterogeneities could lead to the identification of regions of tumor stem cells within regions of lesser proliferative activity known to confer resistance to therapy. Of further importance are those heterogeneous tumor regions parts where the distribution of therapeutic drugs remains low, e.g. in peritoneal or retroperitoneal spread.

We report for the first time a successful high-contrast Cerenkov light imaging following the in vivo application of ^67^Ga isotope. Research measurements and modeling of physical basics and quantum details of effective Cerenkov luminescence using ^67^Ga solutions were outside the scope of our study. However, planning the study we relied on modeling results of Chin and Beattie^23–25^. Building on this modeling work, we assume that the origin of this Cerenkov luminescence is secondary electron spread generation in all tissue water compartments. Looking closer at the radionuclide’s high energy gamma emissions to initiate secondary electrons, we find gamma photons of 17% abundance at 300 keV and 5% abundance near 394 keV and other higher energies of 494, 703, 794, 814 below 0.1% and 888 keV at 0.1%, respectively, as reported in^42,43^. An earlier calculated model of Cerenkov effects^24^ has modeled an availability of 22%: 11 electrons per 50 disintegrations per ^67^Ga-atom decay for the generation of secondary electrons in tissue or water, responsible for Cerenkov photon emission. Beattie^25^ recently reported a more refined model and performed measurements of similarly high-energy gamma rays. Those data have proven the role of secondary electrons in the generation of Cerenkov luminescence photons from isotopes with high-energy gamma emission such as ^67^Ga. A detectable number of photons per disintegration could be present, with slightly below 20% availability of Ga-67 Cerenkov Radiation efficiencies.

^67^Ga-citrate scintigraphy played an important role in lymphoma monitoring earlier and still remains a very important diagnostic means in the majority of the world where PET/CTs are not available to this end. In Low-Income Countries, per million inhabitants, the number of SPECT scanners available is ten-fold compared to PET scanners. In Lower Middle Income, Middle Income and even High Income countries, this proportion is still in the significant range of two to four times the number of SPECT systems to PET^44^. Therefore in countries and in regions without adequate access to PET scanners, ^67^Ga-citrate could still remain an accessible specific radiopharmaceutical for lymphoma staging. The simplicity, low capital investment (circa a tenth to a fifth of any SPECT/CT system price) and low operational costs of CLI devices would provide clinical benefits to lower income countries.

We expected ^67^Ga-citrate accumulation in Bc.DLFL1 lymphoma cells, supposing that ^67^Ga-imaging of lymphoma can also provide information about the staging or monitoring the therapy response. In the clinics, many other studies have already demonstrated that FDG-PET is superior to ^67^Ga-citrate imaging^14^ in agreement with our study. In summary FDG PET has proven to be more sensitive among the two radiopharmaceuticals in this model, as FDG PET and Cerenkov imaging could visualize tumor foci in the abdomen in the advanced stage group of mice, while no such imaging possibility was feasible in the advanced stage group using Ga-67 citrate, only in terminal stage animals. The reason for this can be cell cycle heterogeneity of the tumour tissue cell population. In earlier studies it has been shown that the tumoral cell over-expression of the molecule responsible for Ga-67 cellular uptake, i.e. the transferrin receptor (also referred to as CD71 glycoprotein) is cell-cycle dependent^45,46^. On the other hand, overexpression and functions of glucose transport proteins are less dependent on the cell cycle phases. Theoretically, this offers a higher signal of increased glucose transporter detection in tumors than a Ga-67 uptake based detection relying on CD71 overexpression with fluctuating intensity. Another reason could be that the diagnostic performance of ^67^Ga-citrate imaging is less accurate in case of small foci in the abdomen than in other part of the body due to the high intestinal background activity, but its use is justified in the supradiaphragmatic regions^47–49^. It has been shown in the present mouse lymphoma model study, too, that ^67^Ga-citrate imaging would consequently be less sensitive than FDG-PET imaging. However, ^67^Ga is an efficient therapeutic Auger electron emitter that has been applied in in vitro^50,51^, and also as a radionuclide with therapeutic potential in pre-clinical and preliminary clinical studies^35,45,46^. Using different targeted delivery methods to achieve specific cell nuclear uptake in lymphoma cells and DLBCL patients, ^67^Ga might have a therapeutic potential in lymphoma, too. This could well be applied in conjunction with imaging of biodistribution spaces using CLI.

Our results confirmed the Cerenkov imaging suitability of ^67^Ga-citrate. As ^67^Ga decay emits both gamma radiation and can produce Cerenkov luminescence, it is applicable for both SPECT and CLI. Using this approach, we could locate a distant parathymic lymph node metastasis after intraperitoneal lymphoma injection with intravenous Ga-67 citrate administration followed by SPECT/MRI and ex vivo CLI. This metastatic location is in agreement with earlier demonstration of DLBCL lymphoma spreading following intraperitoneal injection^52^. However, further optimization strategies are needed to explore the full potential of this novel approach. During clinical translation special care has to be taken for hospital staff radionuclide exposure. Observing as low as reasonably achievable (ALARA) radiation protection principles should be paramount regarding staff hazards when designing clinical experiments. Staff ALARA might be the most important challenge to large-scale CLI applications in the clinic.

## Conclusions

These results show that in vivo whole-body imaging with PET or SPECT, followed by ex vivo CLI acquisitions might be guiding tumor resection for pathology evaluation and therapy planning not only in preclinical investigations but for the clinical practice, too. CLI could be applied to outline tumorous tissues and to guide their surgical resection. Ex vivo CLI may guide the pathology sampling of any enlarged lymph nodes that have been previously localised by PET. Other surgically removed lymph nodes can be further investigated ex vivo. Further physics modeling studies and phantom experiments of Ga-67 CLI should be encouraged. In this study context, we report Cerenkov imaging of Ga-67 isotope for the first time. The combined use of SPECT, PET and CLI in clinical oncological practice is warranted.
